# Comparative Genomics Revealed Fluoroquinolone Resistance Determinants and OmpF Deletion in Carbapenem-Resistant *Escherichia coli*

**DOI:** 10.3389/fmicb.2022.886428

**Published:** 2022-04-18

**Authors:** Wan-Ting Yang, I-Ju Chiu, Yao-Ting Huang, Po-Yu Liu

**Affiliations:** ^1^Division of Infection, Department of Internal Medicine, Taichung Veterans General Hospital, Taichung, Taiwan; ^2^Department of Computer Science and Information Engineering, National Chung Cheng University, Chia-Yi, Taiwan; ^3^Ph.D. Program in Translational Medicine, National Chung Hsing University, Taichung, Taiwan; ^4^Rong Hsing Research Center for Translational Medicine, National Chung Hsing University, Taichung, Taiwan

**Keywords:** carbapenem-resistant, whole-genome sequencing, *Escherichia coli*, carbapenemase, virulence, epidemiology

## Abstract

*Escherichia coli* (*E. coli*) is a major causative organism of complicated urinary tract infections, bloodstream infections, and pneumonia. With the widespread use of antimicrobial agents, the prevalence of carbapenem resistance in *E. coli* has been increasing with limited therapeutic options. Fluoroquinolone remains a choice in carbapenem-resistant *E. coli (CREc)* that were once susceptible to the drug. Despite robust studies on the fluoroquinolone-resistant mechanisms of *E. coli*, few studies focused specifically on the group of CREc. In this study, we used comparative genomics to identify the fluoroquinolone-resistant mechanisms of CREc and detected gyrA D87N mutation in all the fluoroquinolone-resistant and *CREc*. Moreover, to investigate the mechanism underlying non-carbapenemase-producing carbapenem-resistant *E. coli*, we targeted the complete genome sequences for in-depth analysis and found a deletion in OmpF (DEL264-269) that might contribute to carbapenem resistance, which has not been reported before. Further studies focusing on the impact of these mutations on the expression levels are warranted. We further investigate the MLST, serotype, fimH type, phylogroup, and clinical characteristics of the *CREc*. Combination analysis of clinical and genomic characteristics suggests the polyclonal and highly diverse nature of the *CREc* in Taiwan. This study provides an insight into the molecular epidemiology of CREc in Taiwan.

## Introduction

*Escherichia coli* (*E. coli*) is a major causative organism of complicated urinary tract infections, bloodstream infections, and pneumonia. Due to the advent of antimicrobial resistance, therapeutic options have become limited. The carbapenems are sometimes the only effective regimens against the severe infection caused by drug-resistant *E. coli*. However, with the widespread use of antimicrobial agents, the prevalence of carbapenem resistance in *E. coli* has increased. The reported occurrence of carbapenem-non-susceptible or -resistant *E. coli* varies by region. The prevalence rate of carbapenem-non-susceptible *E. coli* was 0–3% among the 12 Asia-Pacific nations (Mendes et al., 2013), 4.3% among hospitals in the US (McCann et al., 2018; Castanheira et al., 2020), and 0–7% in Europe (Kostyanev et al., 2019). According to surveillance conducted in Taiwan in 2009, the rate of carbapenem non-susceptibility in *E. coli* was 1.6–7.1% (Jean et al., 2013). The proportion of carbapenem-non-susceptible and -resistant *E. coli* has been increasing (Martirosov and Lodise, 2016). Moreover, patients infected with carbapenem-resistant pathogens, when compared to those infected with susceptible pathogens, showed increased morbidity and mortality (Nordmann and Poirel, 2019). The crude mortality of patients with carbapenem-resistant Enterobacterales infections is estimated to be 70% (Friedman et al., 2017). Carbapenem resistance increases the medical cost and causes an enormous economic burden (Bartsch et al., 2017).

Fluoroquinolone remains a choice in carbapenem-resistant *E. coli* (CREc) if the bacteria is found to be susceptible to the drug. Despite robust studies on fluoroquinolone-resistant mechanisms of *E. coli*, few studies specifically addressed the CREc group.

Two important mechanisms of resistance are observed in CREc. One is mediated by the carbapenemase genes, and the isolates are termed as carbapenemase-producing carbapenem-resistant *E. coli* (CP-CREc). They are classified according to the Ambler classification into three groups: class A, class B, and class D enzymes (Ambler, 1980). The mechanism of resistance in non-carbapenemase-producing carbapenem-resistant *E. coli* (non-CP-CREc) is mediated by ESBL or AmpC beta-lactamase associated with an overexpression of efflux pumps (such as AcrAB) or a loss of expression of porin (such as OprD and OprM) (Chetri et al., 2019; Theuretzbacher et al., 2020).

The prevalence of CP-CREc has increased in recent years in Taiwan (Jean et al., 2013), and the reported types of carbapenemases in CP-CREc is also increasing (Dortet et al., 2017). However, some mechanisms that are responsible for carbapenem resistance in *E. coli* are not well understood. In addition, the phylogenetic relationship, clinical phenotypes, and resistome characteristics still warrant further investigation. Whole-genome sequencing (WGS) provides a comprehensive method to identify the mechanisms underlying carbapenem resistance in *E. coli*, in particular non-CP-CREc, such as porin-encoding gene alternation along with ESBL or AmpC (Larkin et al., 2020). In this study, we aim to use WGS to characterize the molecular resistance mechanisms, virulence determinants, and phylogenetic relatedness of CREc.

## Methods

### Bacterial Isolates and Antibiotic Susceptibility Testing

This study was conducted at a tertiary hospital in Taichung, Taiwan. The CREc samples were collected from patients with bacteremia admitted during the period from January 2015 to May 2019, and we randomly selected five quinolone-resistant *E. coli* and five quinolone-susceptible *E. coli*. The *E. coli* isolates resistant to any of the ertapenem (minimum inhibitory concentrations [MICs] of ertapenem ≤ 2 μg/ml), imipenem, or meropenem (MIC of imipenem and meropenem ≤ 4 μg/ml) derivatives were enrolled in the study. We used MALDI-TOF (matrix-assisted laser desorption ionization-time of flight) mass spectrometry analysis for the identification of bacteria. The MICs of gentamicin, amikacin, ceftriaxone, ceftazidime, cefepime, piperacillin-tazobactam, ertapenem, imipenem, ciprofloxacin, and trimethoprim-sulfamethoxazole were determined by using VITEK 2 Automated System (BioMe rieux, Marcy l'Etoile, France). We used Clinical and Laboratory Standards Institute-established criteria (CLSI M100-S29) to determine the susceptibility of bacteria to each antibiotic. Susceptibility to ceftazidime-avibactam was determined by broth microdilution method, as suggested by the Clinical and Laboratory Standards Institute (CLSI).

### Epidemiological and Clinical Characteristics

We retrospectively reviewed the medical files of the patients with positive isolates of CREc. We collected demographic data, including age, gender, underlying conditions, and the use of antibiotics. In addition, antimicrobial susceptibility results, clinical parameters, and outcomes were also collected. The Pitt bacteremia score (PBS) is a severity of acute illness index and is broadly used in studies related to bloodstream infections. It scores from 0 to 14 points, and score ≥4 denotes critical condition and increased mortality (Chow and Yu, 1999). The sources of bacteremia were determined by a combination of clinical, radiological, and laboratory investigations. Intra-abdominal infection (IAI) encompasses a number of infectious processes, including peritonitis, cholecystitis, liver abscess, and bacterial translocation. Community-acquired CRE isolates were collected within 48 h of the patient's admission to the hospital. Hospital-acquired CRE isolates were collected after 48 h of admission (Garner et al., 1988; Weinstein et al., 1997).

### DNA Extraction, Whole-Genome Sequencing, and Assembly of Genomes

Ten isolates of CREc were subjected to WGS using Nanopore GridION. The DNA library was prepared using the rapid barcoding kit (SQK-RBK004) by following the manufacturer's instructions, and the sequencing was performed in a R9.4.1 flowcell for 48 h. The sequenced reads were basecalled using Guppy 4.4.2. The adaptor sequences left in the reads were trimmed using Porechop (v0.2.4). The clean reads were assembled into contigs *via* Flye (v2.7) (Kolmogorov et al., 2019), and the remaining sequencing errors were sequentially polished by Racon, Medaka, and Homopolish (Vaser et al., 2017; Huang et al., 2021b). The assembled contigs were classified into chromosomes and plasmids using both NCBI BLAST and PlasmidFinder. Protein-coding genes, coding and non-coding RNAs in the chromosomes, and plasmids were annotated using the NCBI Prokaryotic Genome Annotation Pipeline (PGAP) (Tatusova et al., 2016). Antibiotic-resistant genes (ARGs) were predicted by aligning protein-coding genes against the Comprehensive Antibiotic Resistance Database (CARD) (Alcock et al., 2019). Only ARGs with alignment coverage greater than 90 % were retained. Efflux pumps were excluded from the ARG analysis. Beta-lactamases were separately predicted by using the curated hidden Markov models in NCBI AMRFinderPlus (Feldgarden et al., 2021).

The phylogeny of two outer membrane proteins, OmpC and OmpF, was reconstructed by MEGA (v11) and visualized by the Interactive Tree of Life (ITOL) (Tamura et al., 2021). Point mutations, insertions, and deletions in these porins were manually identified from the multiple sequence alignment in MEGA. Mutations within the quinolone resistance-determining region in GyrA, GyrB, ParC, and ParE of each CREc isolate were found by BLAST alignment against each other. The protein effects of each mutation (i.e., neutral or deleterious) were assessed by PROVEN (Choi et al., 2012).

### Phylogenetic Grouping, MLST, Serotyping, and Virulence Determinant Analyses

We uploaded the assembled genomes into the Center for Genomic Epidemiology (http://www.genomicepidemiology.org/), and the plasmid replicon type, MLST, serotype, and *fim*H type were determined using the following programs: PlasmidFinder (version 2.0.1), MLST (version 2.0.4), SerotypeFinder (version 2.0.1), VirulenceFinder 2.0 (version: 2.0.3), and *Fim*Typer (version 1.0), respectively (Larsen et al., 2012; Carattoli et al., 2014; Joensen et al., 2014; Roer et al., 2017; Malberg Tetzschner et al., 2020). We uploaded the assembled genomes to the ClermonTyping v1.4.0 web tool to assess the phylogroups (Alikhan et al., 2018; Beghain et al., 2018).

## Results

### Demographic and Genetic Data

The clinical characteristics are summarized in Table 1. An equal number of strains were isolated from men and women. The median age of the patient was 66.5 years (IQR = 60–80). Half of the isolates suffered from community-acquired bacteremia, while the other half contracted hospital-acquired bacteremia. The sources of bacteremia in five isolates were urinary tract, while the intra-abdominal infection was the source of bacteremia in the remaining five isolates. Two patients expired, one of them was diagnosed with spontaneous bacterial peritonitis and the other had a liver abscess. Six out of the 10 patients were exposed to a carbapenem and broad-spectrum cephalosporin in the previous year. The median of the PBS was 0.5 (IQR = 0–3). Elppa 4, which harbored OXA-48 carbapenemase, had the highest PBS 12, and the second-highest PBS 7 was found in Elppa 8, which harbored NDM-1 mutation. Three out of 10 CREc isolates were carbapenemase-producing bacteria (OXA-48, *n* = 2; NDM-1, *n* = 1). Among non-CP-CREc, four out of seven isolates have ESBL or AmpC beta-lactamase. Comparative genomics revealed *OmpC* D192G mutation in four isolates and *OmpF* 264-269 deletion in another three isolates.

**Table 1:** Demographic and genetic data for the 10 *E. coli* isolates from Central Taiwan.

	**Strain ID**	**Age**	**Sex**	**Underlying disease**	**Antibiotics usage in prior year**	**Source of bacteremia**	**Outcome**	**The pitt bacteremia score**	**Carbape** **nemase**	**ESBL, Ampc**	**Porin**
Community-acquired bacteremia	Elppa1	80	M	HTN	LVX	UTI	Survived	0			*Omp*C D192G mutation
	Elppa2	66	F	No underlying disease	None	Acute cholecystitis	Survived	1			*Omp*F 264-269 deletion
	Elppa8	69	M	CJD	CEX	UTI	Survived	7	NDM-1		
	Elppa9	47	F	HBV carrier, fatty liver	None	Acute cholecystitis	Survived	0		CMY-2	*Omp*F 264-269 deletion
	Elppa10	93	F	Type A aortic dissection, CKD stage IV	None	UTI	Survived	1			*Omp*C D192G mutation
Hospital-acquired bacteremia	Elppa3	45	M	PCKD, CKD stage V	FLO	UTI	Survived	0		CMY-2, CMY-29	*Omp*C D192G mutation
	Elppa4	81	F	Cervical cancer, radiation cystitis	CFPM	UTI	Survived	0	OXA-48		
	Elppa5	62	M	CAD, DM	None	Spontaneous bacterial peritonitis	Died	12	OXA-48		*Omp*F 264-269 deletion
	Elppa6	60	M	HCC, HBV, duodenal bleeding	ETP, Cefo/sulb	Liver abscess	Died	3		CMY-2, CTX-M	*Omp*C D192G mutation
	Elppa7	67	F	HCV, liver cirrhosis, PCKD, ESRD	DOR, CAZ	IAI	Survived	0		CMY-2, CTX-M-5	

*CAD, coronary artery disease; CAZ, ceftazidime; Cefo/sulb, Cefoperazone/sulbactam; CFPM, cefepime; CEX, cephalexin; CKD, chronic kidney disease; CJD, Creutzfeldt-Jakob disease; DM, diabetes mellitus; DOR, doripenem; ESRD, end-stage renal disease; ETP, ertapenem; FLO, flomoxef; HBV, hepatitis B virus; HCC, hepatocellular carcinoma; HCV, hepatitis C virus; HTN, hypertension; LVX, levofloxacin; PCKD, polycystic kidney disease; UTI, urinary tract infection*.

### Mechanism of Carbapenem Resistance in *E. coli*

Among the 10 CREc isolates, three were carbapenemase-producing *E. coli* (CP-CREc, *n* = 3/10), with two harboring OXA-48 gene and one harboring NDM-1 gene, as presented in Table 2. None of the CP-CREc isolates had ESBL or AmpC enzymes. The OXA-48 genes are located on plasmids, and the NDM-1 gene is located on the chromosome. Seven isolates had no known carbapenemase genes. Among these non-CP-CREc, two isolates harbored AmpC enzymes (Elppa 3 and 9), and two isolates held the combination of both ESBL and AmpC (Elppa6 and 7). In comparison, three isolates neither had AmpC nor ESBL but had only broad-spectrum beta-lactamase (Elppa 1, 2, and 10).

**Table 2:**
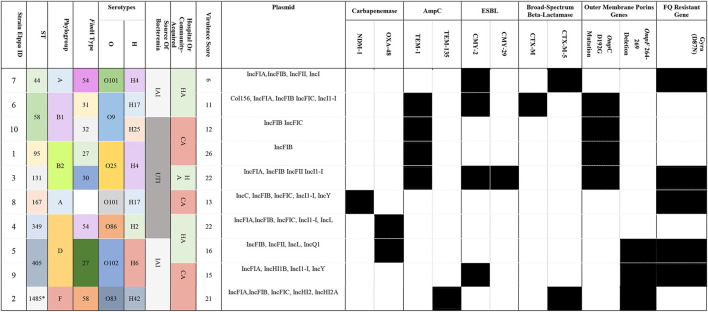
Genetic diversity, source of bacteremia, and antibiotic-resistant mechanisms identified in the *E. coli* strains.

*Virulence score is the number of virulence genes detected in an isolate. CA, community-acquired; FQ, fluoroquinolone; HA, hospital-acquired; IAI, intra-abdominal infection; ST, sequence type; UTI, urinary tract infection*.

*The black shade indicate present and color shade indicates Marks of typing*.

**Figure 1:**
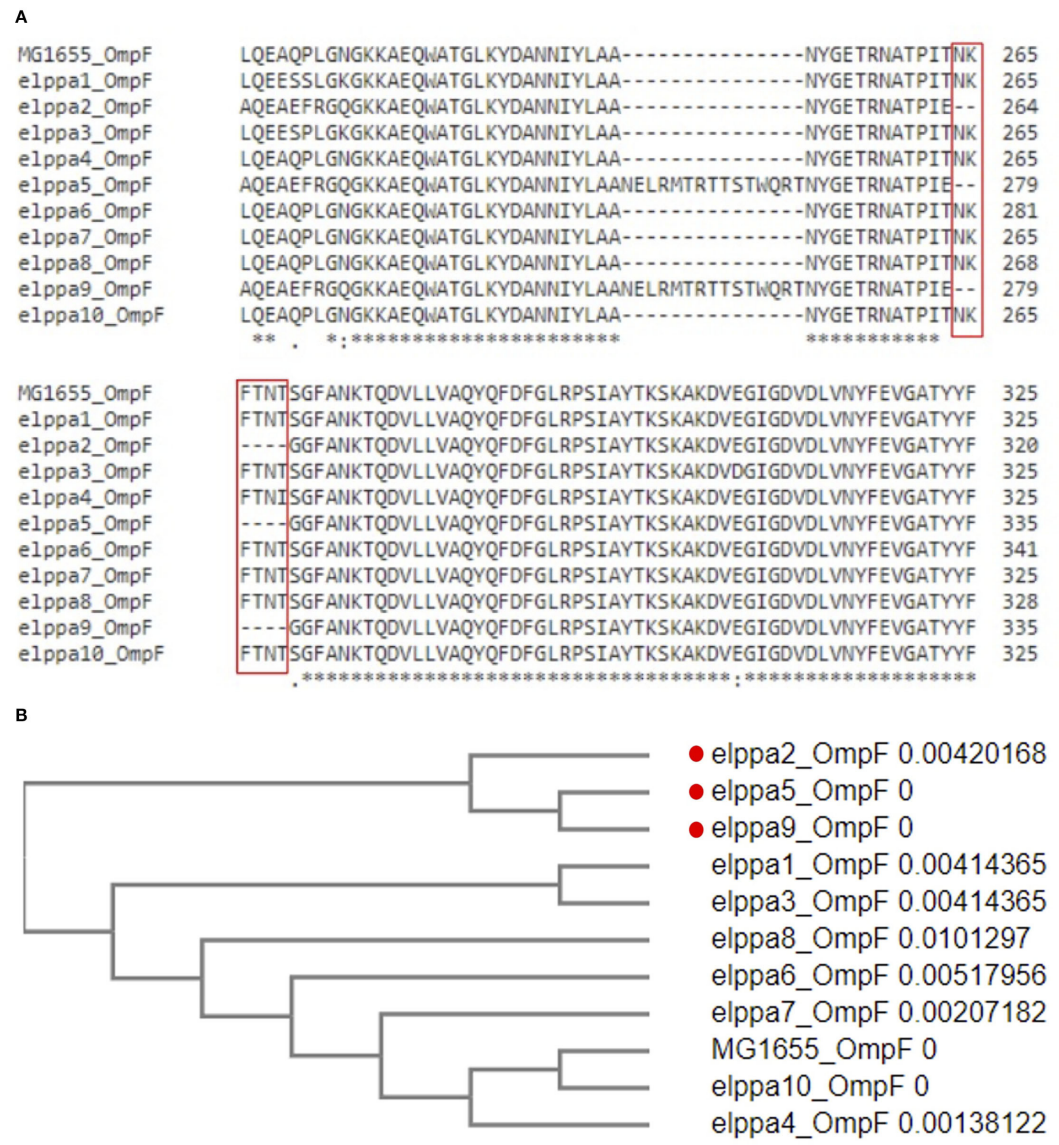
**(A)** Multiple sequence alignment of OmpF revealed deletion in 264-269. **(B)** Phylogenetic clusters in deleted OmpF.

Loss of porin can also contribute to carbapenem resistance. In addition to the known *OmpC* D192G mutation (e.g., Elppa 1, 3, 6, and 10), we identified a novel deletion in *OmpF* (DEL264-269) in three isolates (Elppa 2, 5, and 9) (Figure 1A), where two of them lack carbapenemase. These carbapenemase-negative isolates may contribute to carbapenem resistance *via* loss of OmpF in conjunction with other beta-lactamases (e.g., TEM in Elppa 2 and CTX-M in Elppa 9). In particular, we observed that Elppa 2, 5, and 9 are phylogenetically clustered in OmpF (Figure 1B), implying that a subgroup resistant to carbapenems using a new mechanism is emerging. Similarly, the three isolates carrying the D192G mutation in *Omp*C are also grouped together (Supplementary Figure 1). These results suggested that multiple pathways leading to carbapenem resistance are emerging in these isolates.

### Antimicrobial Resistance Profile and Resistance Mechanism Among *CREc* Isolates in This Study

Supplementary Table 1 presents the susceptibility testing results of CREC to antimicrobial agents. All the CREc isolates were resistant to ceftriaxone, ceftazidime, cefepime, piperacillin-tazobactam, ertapenem, and imipenem. The susceptibility rate for ciprofloxacin was 50%, in concordance with the isolation of *gyrA* (D87N) mutation (Table 2). The only isolate resistant to ceftazidime-avibactam was Elppa8, which had NDM-1 mutation.

We further compared the susceptibility patterns to different antimicrobial agents between non-carbapenemase-producing and carbapenemase-producing carbapenem-resistant *E. coli* isolates, and the results are presented in Table 3. The susceptible rate of ciprofloxacin (CP-CREc vs. Non-CP-CREc, 33.3 vs. 57.1%), amikacin (100 vs. 66.7%), and ceftazidime-avibactam (100 vs. 66.7%) was lower in CP-CREc when compared to Non-CP-CREc isolates.

**Table 3:** The susceptibility patterns to different antimicrobial agents between non-carbapenemase-producing and carbapenemase-producing carbapenem-resistant *E. coli* isolates.

**Carbapenem-resistant** ***E. coli*** **(%Susceptible)**			
**Antimicrobial agents**	**All (*****n =*** **10)**	**Non-CP-CREc** **(*****n =*** **7)**	**CP-CREc** **(*****n =*** **3)**
Ceftriaxone	0	0	0
Ceftazidime	0	0	0
Cefepime	0	0	0
Ceftazidime-avibactam	90	100	66.7
Amikacin	90	100	66.7
Gentamicin	20	14.3	33.3
Ciprofloxacin	50	57.1	33.3
Pip/Tazo	0	0	0
TMP/SMX	30	28.6	33.3
Ertapenem	0	0	0
Imipenem	0	0	0

*Non-CP-CREc, non-carbapenemase-producing carbapenem-resistant E. coli; CP-CREc, carbapenemase-producing carbapenem-resistant E. coli*.

### Phylogenetic Grouping, MLST, and Serotyping

Different typing approaches were used to unravel population structure and genetic diversity, including MLST, phylogenetic grouping, and serotyping, presented in Table 2. The CREc strains in this study showed a great variation in the sequence type, and eight ST types were identified. ST 58 and ST405 were detected in two isolates, while the remaining were singletons. The three carbapenemase-producing strains belong to different ST types. The *E. coli* isolate carrying NDM-1 gene was categorized as ST167, and the two OXA-48-positive *E. coli* strains were classified as ST 349 and ST 405, respectively. The ST types of non-carbapenemase-producing *E. coli* were also highly diverse. This finding implicates the polyclonal pathogenic carbapenem-resistant *E. coli* population.

In addition, we detected five phylogenic groups. Three isolates belonged to phylogroup D and two belonged to phylogroups B1 and B2. Phylogroup B2, which is in line with phylogroup D, was reported to include virulent extra-intestinal strains (Chakraborty et al., 2015). Phylogroups B2, F, and D had the highest number of virulence factors, with an average of 24, 21, and 17, respectively.

### Virulence Genes

In total, we identified 47 virulence genes, and these genes impact different aspects of functions in *E. coli*, including adherence, autotransporter, enzyme, invasion, iron acquisition, secretion system, and toxin generation, which is presented in Table 4. The maximum number of detected virulence genes was 26 in Elppa1, which belonged to phylogroup B2. Elppa 7 had the smallest number of virulence genes (*n* = 6), and it belonged to phylogroup A.

**Table 4:**
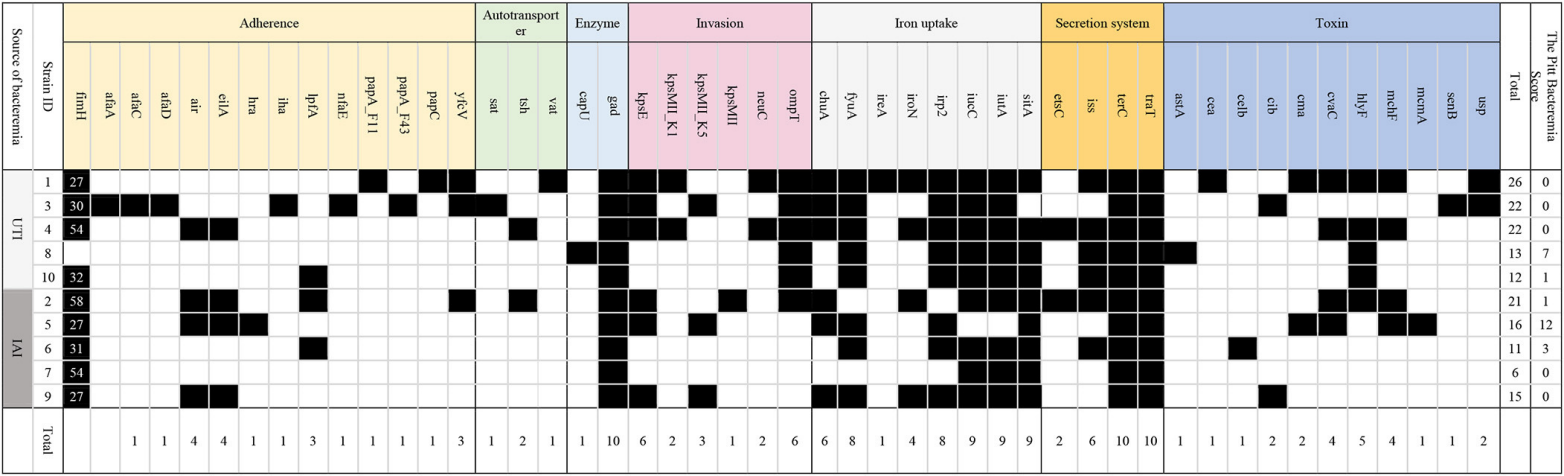
Heatmap presents in silico analyses of virulence genes.

*A Black shaded box indicates gene is present. For fimH, numbers within shaded cells denote allelic variants*.

Among the adhesion proteins of pathogenic *E. coli*, type 1 fimbrin D-mannose specific adhesin protein plays an important role and is encoded by the *fim*H gene. *Fim*H typing provides a method for epidemiological typing of pathogenic *E. coli*. In this study, six types of *fim*H gene were identified in nine isolates, with *fim*H27 being the commonest (*n* = 3).

**Figure 2:**
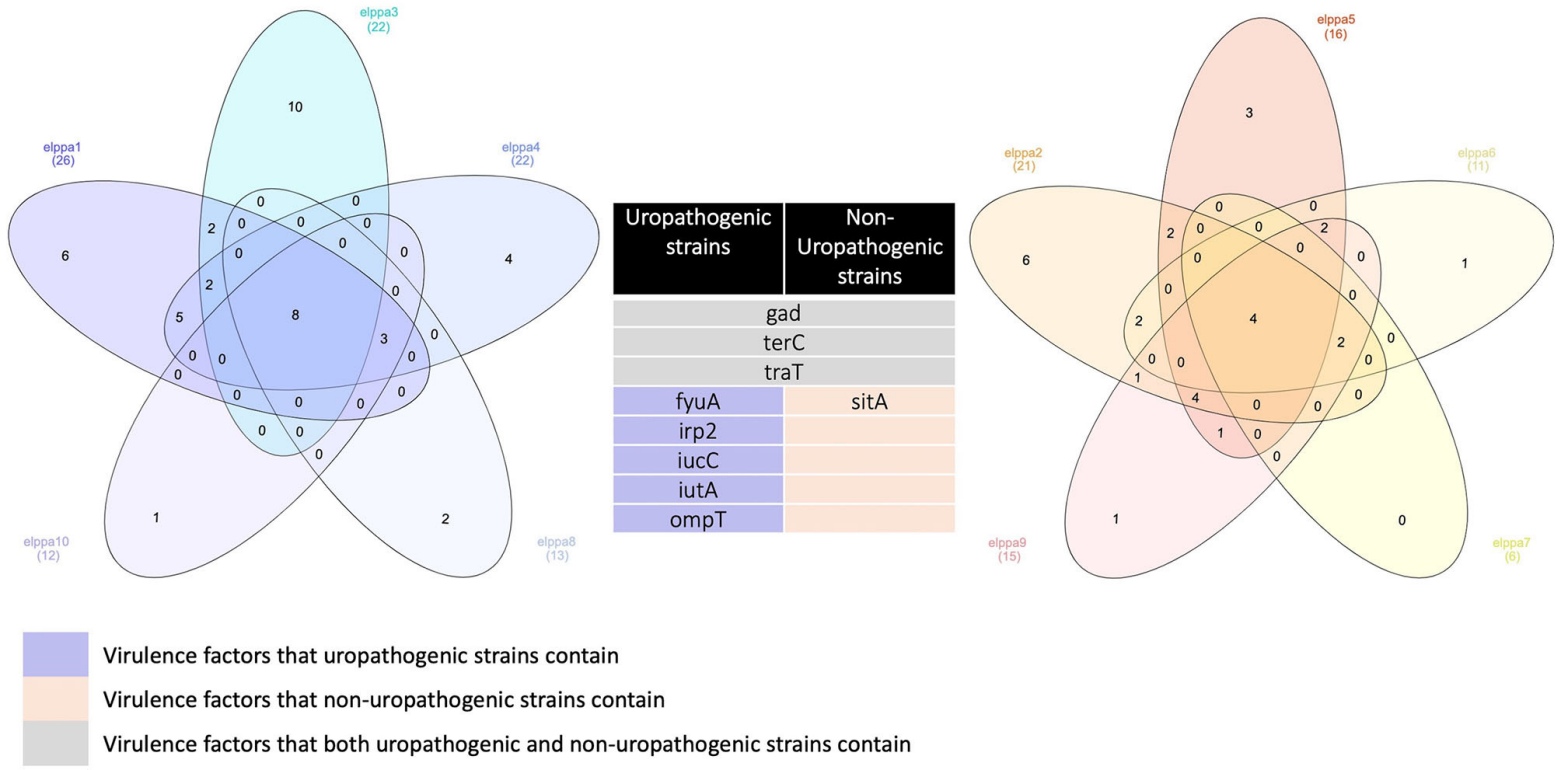
Venn diagrams comparing virulence factors of bloodstream infections from abdominal origin or urologic origin. Five *E. coli* isolates from the urinary tract shared eight virulence factors, including *gad, terC, traT, fyuA, irp2, iucC, iutA*, and *ompT*. Five *E. coli* isolates from abdominal origin shared four virulence factors, including *gad, terC, traT, and sitA*. Bloodstream infection from both sources contained virulence factors of *gad, terC*, and *traT*. Venn diagrams were created by InteractiVenn (Heberle et al., 2015).

The most common genes that are sequenced are genes linked to adherence and toxin. All *E. coli* isolates in the present studies harbor *gad, terC*, and *traT* (Figure 2), which are all protection factors, including genes that mediate resistance to substances harmful to bacterial growth and are associated with the capsule. Glutamate decarboxylase (gad) is one of the most effective acid resistance systems in *E.coli*.

The expression of glutamate decarboxylase is induced to maintain the physiological pH under acidic conditions (Capitani et al., 2003). *Gad* is particularly relevant for enteropathic *E. coli*, but it has been identified in patients with UTI, IAI, and bacteremia (Chen et al., 2021). Tellurite is deleterious to most bacteria. Tellurium ion resistance (*TerC*) has been implicated in tellurium resistance. It is hypothesized to promote the efflux of tellurium ions (Nguyen et al., 2021). *TraT* is an outer membrane protein that hinders the formation of membrane attack complex in the later stages (Miajlovic and Smith, 2014).

We further compared the virulence factors between uropathogenic and non-uropathogenic *E. coli* strains. Uropathogenic *E. coli* strains were the strains that recovered from the urinary sources of bacteremia, while non-uropathogenic *E. coli* strains were those that recovered from the intra-abdominal source. Uropathogenic *E. coli* strains (*n* = 5, totally contain 95 virulence factors) when compared to non-uropathogenic *E. coli* strains (*n* = 5, totally contain 69 virulence factors) had higher frequencies of virulence genes, as presented in Table 4 and Figure 2. All the uropathogenic strains had *fyuA, irp2, iucC, iutA, ompT. FyuA, irp2, iucC*, and *iutA* genes, and all were related to iron uptake.

To increase virulence, all the extra-intestinal pathogenic *E. coli* strains harbor siderophores, such as enterobactin, salmochelin, yersiniabactin, and aerobactin (Sarowska et al., 2019). *Irp*2 and *fyu*A were associated with yersiniabactin (Schubert et al., 1998), while *iuc*C and *iut*A were associated with aerobactin (Ling et al., 2013). In addition, all the non-uropathogenic strains discovered from intra-abdominal infection harbor *SitA. SitA* is a subunit of an ABC-type ferric iron transport system (Reigstad et al., 2007), which mediates iron uptake from the periplasm into the cytoplasm.

## Discussion

Carbapenems are the last resort to treat multidrug-resistant Gram-negative *E. coli*. With the increasing use of carbapenems, the prevalence of carbapenem-resistant *E. coli* is growing and poses a threat to public health and challenges to physicians. Only a few antibiotics are left for physicians to treat severe infectious diseases (Huang et al., 2021a). An understanding of the resistance mechanisms is essential to therapy and improved control measures. In this study, we demonstrated a high level of genetic diversity in CREc in both community and hospital settings. A previous study found that 0.04–29.5% of the CRE isolates were associated with the community (Kelly et al., 2017). Another report from Taiwan identified that 29.5% of CRE cases were community-acquired infections (Tang et al., 2016). In line with this study, CRE can be acquired from the community and hence poses an urgent public health threat.

**Figure 3:**
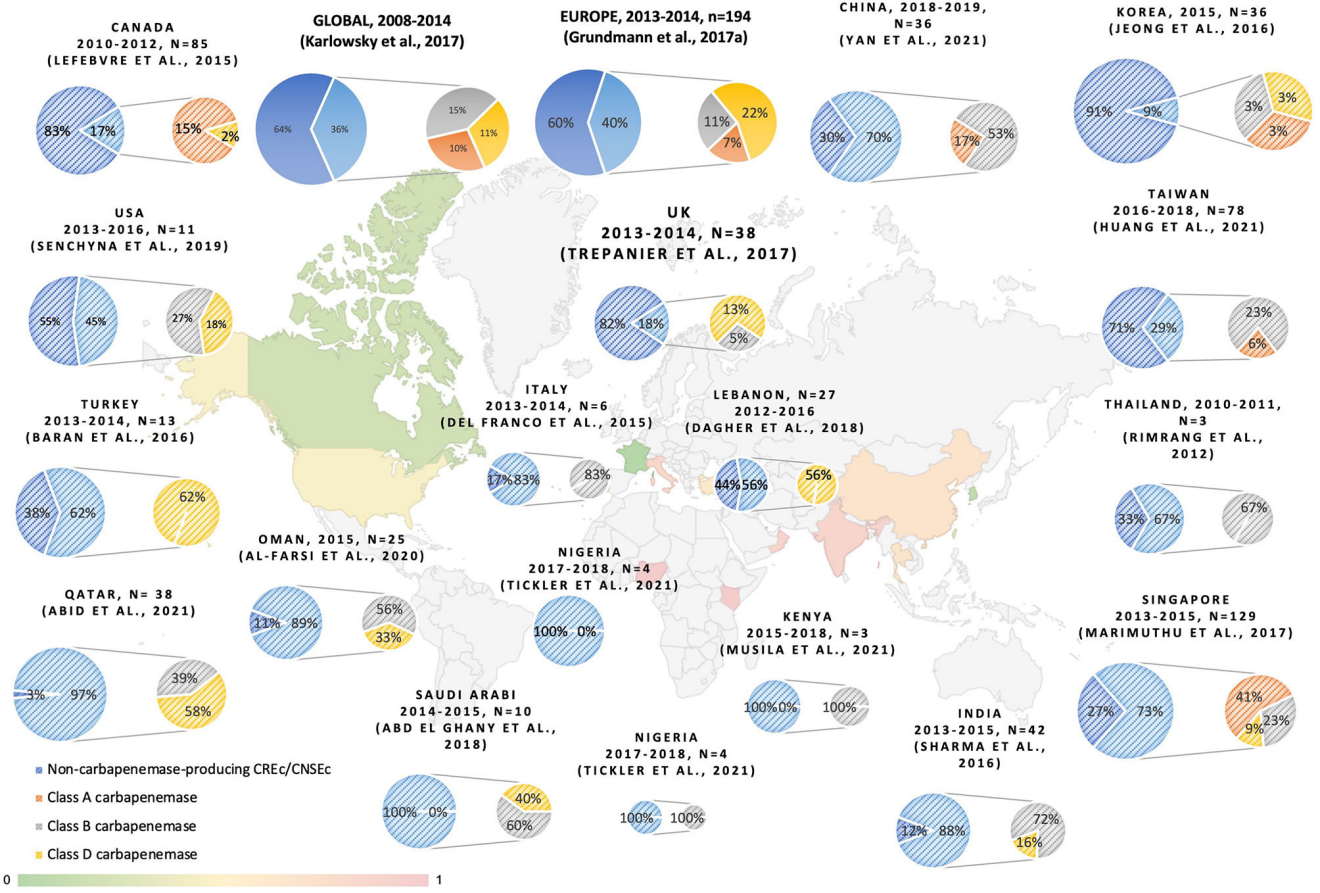
The reporting proportion of non-CP-CRE and carbapenemase among CREc/CNSEc (Rimrang et al., 2012; Del Franco et al., 2015; Lefebvre et al., 2015; Baran and Aksu, 2016; Dupont et al., 2016; Jeong et al., 2016; Sharma et al., 2016; Grundmann et al., 2017; Karlowsky et al., 2017; Marimuthu et al., 2017; Trepanier et al., 2017; Abd El Ghany et al., 2018; Dagher et al., 2018; Senchyna et al., 2019; Al-Farsi et al., 2020; Abid et al., 2021; Huang et al., 2021a; Musila et al., 2021; Tickler et al., 2021; Yan et al., 2021).

In our study, the majority of CREc are not non-carbapenemase-producing strains. Two main molecular mechanisms contribute to carbapenem resistance in Enterobacteriaceae. The resistance is mediated either by carbapenemase production or by ESBL or AmpC in combination with increased efflux pump activity or porin alternation, as observed in the cases of non-carbapenemase-producing CRE. Among carbapenem-resistant and non-susceptible *E. coli*, the proportion of carbapenemase-producing strains varies across different regions of the world, as shown in Figure 3. Despite the fact that the definition of carbapenem non-susceptibility or resistance slightly varied between the studies, a trend toward an increasing number of CP-CREc cases is observed. The predominant carbapenemase varies in different geographic areas, as shown in Figure 3. Among the *E. coli* isolates that were non-susceptible/resistant to carbapenem in Taiwan, the rate of CP-CREc occurrence increased from 1.4% during 2010–2012 (Wang et al., 2015), to 7.59% during 2012–2015 (Chang et al., 2019), and to 29.5% during 2016–2018 (Huang et al., 2021a). According to previous studies in Taiwan, NDM-1 is the most common carbapenemase and accounts for 39.1% of CP-CREc (Chang et al., 2019; Huang et al., 2021a). In this study, 30% of the CREc are CP-CREc. Among the CP-CREc strains, OXA-48 and NDM-1 were presented. The data provide insight into the molecular characteristic of CREc in Central Taiwan.

In this study, we detected *OmpC* D192G and *OmpF* 264-269 deletions using comparative genomics in non-CP-CREc. Porins, which are outer membrane proteins, block antibiotic permeation into bacteria and contribute to antibiotic resistance. Mutation in porins mediates antibiotic resistance by three different mechanisms: an alteration in the electrostatics of the channel, a reduction in the size of the porin channel, or a decrease in the expression level of porins or loss of porins (Fernández and Hancock Robert, 2012). In *E. coli, two* modifications of porin were observed: loss of OmpC and OmpF and mutation in OmpC (Vergalli et al., 2020). Lou et al. (2011) and Bajaj et al. (2016) proved that a single mutation in OmpC proteins could decrease the permeability of an antibiotic, by using liposome permeation assays and single-channel electrophysiology. *OmpC* D192G has been identified in a previous study and verified by PROVEAN (Al-Farsi et al., 2020; Tian et al., 2020). To our best knowledge, *OmpF* 264-269 deletion has not been reported before. *OmpF* 264-269 deletion might contribute to carbapenem resistance. Further studies regarding the impact of these mutations on the expression level are warranted.

In the present study, 10 isolates belonged to eight different ST types, indicating polyclonal spreading. Previous studies reported that 55.6% of NDM-positive *E. coli* isolates were classified as ST410 type (Huang et al., 2021a), and among carbapenem non-susceptible *E. coli*, ST131 is the predominant ST type in Taiwan (Ma et al., 2013; Mathers et al., 2015). The NDM-1-producing *E. coli* in the present study belonged to ST167, which differed from the results of previous studies. These findings suggest that pathogenic *E. coli* in Taiwan is polyclonal and highly genetically diverse.

The NDM-1 gene identified in this study is located within the chromosome, and the isolate belongs to ST167. Usually, *bla*_NDM_ genes are located in various types of plasmids, such as IncA/C, IncF, IncNIncL/M, and untypable/IncR (Poirel et al., 2011; Khan et al., 2017). Shen et al. reported the first case of a chromosomally integrated *bla*_NDM−1_ *E. coli* which originated in China. The *bla*_NDM−1_ gene was inserted between two tandem copies of an insertion sequence common region 1 element, which originated from the plasmid of *Proteus mirabilis*. The chromosomally encoded *bla*_NDM−1_ *E. coli* strain belonged to ST167, which is identical to the *bla*_NDM−1_ *E. coli* strain of this study (Shen et al., 2017). In this study, we demonstrated that *bla*_NDM−1_ in *E. coli* can be chromosomally integrated and transmitted vertically. Close surveillance and monitoring are warranted to prevent the spread of the *bla*_NDM−1_ chromosomally integrated *E. coli* strains.

We also searched for the mechanism of quinolone resistance among the five quinolone-resistant CREc, and no qnr-like, horizontally transferred resistant genes were detected by BLAST in all the plasmids. We found GyrA D87N mutation within the chromosome in all the quinolone-resistant CREc strains in this study.

**Figure 4:**
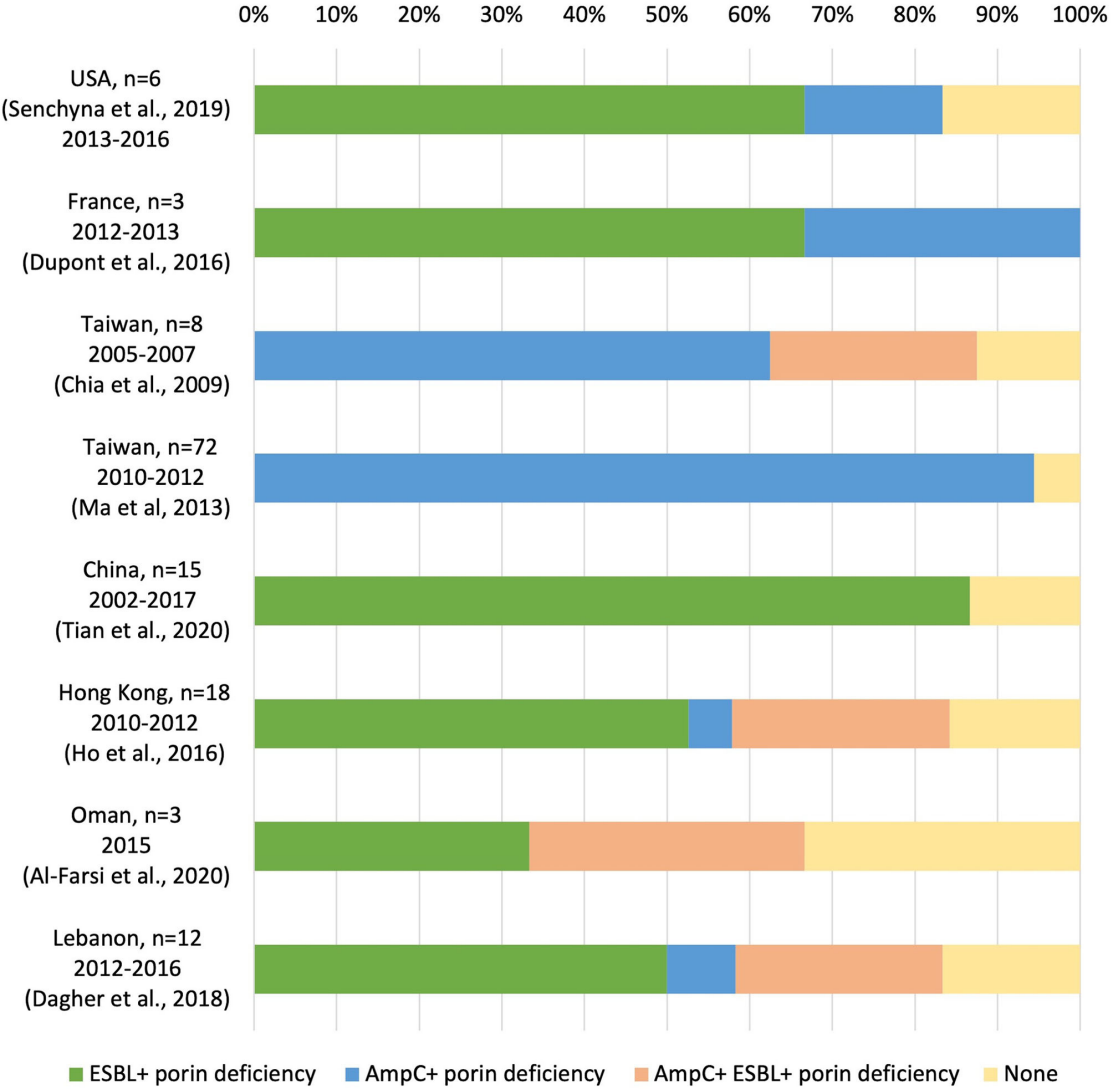
Molecular resistance mechanisms in non-carbapenemase-producing carbapenem-resistant *E. coli*/carbapenem non-susceptible *E.coli* (non-CP-CREc /non-CP-CNEc) (Chia et al., 2009; Ma et al., 2013; Dupont et al., 2016; Ho et al., 2016; Dagher et al., 2018; Senchyna et al., 2019; Al-Farsi et al., 2020; Tian et al., 2020).

Whole-genome sequencing is an emerging technology to investigate molecular resistance mechanisms. While most previous studies used PCR to identify ESBL and AmpC beta-lactamase (Chia et al., 2009; Dupont et al., 2016; Ho et al., 2016; Tian et al., 2020), some studies employed WGS to identify molecular resistant mechanisms in non-carbapenemase-producing CREC (non-CP-CREc)/carbapenem non-susceptible *E.coli* (non-CP-CNEc) (Dagher et al., 2018; Senchyna et al., 2019; Al-Farsi et al., 2020). The major mechanisms of the non-CP-CRE/CNEC are AmpC or ESBL β-lactamase in combination with alternation or loss of an outer membrane porin (OmpC and OmpF), as shown in Figure 4. In Taiwan, plasmid-encoded AmpC β-lactamase CMY-2 and DHA-1 combined with OmpC/F loss was the primary mechanism (Chia et al., 2009; Ma et al., 2013). Despite intensive surveys, an average of 16% of the non-CP-CREc/CNEc isolates in the literature cannot be fully explained by the above-mentioned mechanism. In the present study, we found *OmpF* 264-269 deletion and verified *via* the online PROVEAN platform that this deletion was deleterious. There are still knowledge gaps regarding the mechanism underlying non-CP-CREc. Further studies on the efflux pump and regulatory protein, which regulate the expression of outer membrane porin, may improve our understanding of these issues.

A previous study demonstrated that CP-CRE strains had greater potency to hydrolyze carbapenems than non-CP-CRE strains and were more likely to resist other non-β-lactam antibiotics (Tamma et al., 2017). However, in our study, we could not identify differences in the MIC distribution of carbapenems. We did find that the susceptible rate of ciprofloxacin was lower in CP-CREc (33.3%) than in Non-CP-CREc (57.1%). Previous studies have proved that the mode of CREc acquisition differs between CP-CREc and non-CP-CREc isolates. The mode of acquisition of non-CP-CREc was through an endogenous pathway, which involves selective pressure of antibiotics on the intestinal microbiota and is primarily associated with recent exposure to antibiotics. While for CP-CREc, the mode of acquisition was primarily exogenous through “patient-to-patient” horizontal spread (Goodman et al., 2016; van Duin and Doi, 2017; Bouganim et al., 2020). By investigating the molecular mechanisms of CREc, we can better predict the risk factors among patients who are at risk of being infected with CREc and prevent CREc.

All *E. coli* strains in the present study harbor *gad, terC*, and *traT*. In this study, we found that uropathogenic *E. coli* strains (*n* = 5, totally contain 95 virulence factors), compared with non-uropathogenic *E. coli* strains (*n* = 5, totally contain 69 virulence factors), had higher frequencies of virulence genes. Extra-intestinal pathogenic *E*. *coli* (ExPEC) are multifaceted pathogens that can cause infections at extra-intestinal sites. The ExPEC group includes avian pathogenic *E. coli*, neonatal meningitis *E. coli*, uropathogenic *E. coli* (UPEC), and sepsis-associated *E. coli* (SEPEC). ExPEC are pathogenic to humans due to the presence of a constellation of virulence genes. A previous study discovered that *E. coli* that caused IAIs had fewer virulence genes than those that caused UTIs or primary bacteremia (Chen et al., 2021). It was postulated that during IAI, enteric *E. coli* may inadvertently translocate from the intestine to the bloodstream. A previous study had suggested that CP-CRE may be more virulent than non-CP-CRE and are associated with poorer outcomes (Tamma et al., 2017). Analysis has indicated zoonotic origin for ExPEC, as evident by the fact that avian and human *E. coli* isolates encompass similar virulence genes and that they belong to the same phylogenetic groups. This finding reminds us that to control the spread of ExPEC, poultry product surveillance and monitoring are important steps (Sarowska et al., 2019).

This study has several limitations. First, a genotype is not equal to a phenotype. Isolates harboring carbapenemase-producing genes are not identical to the isolates that express carbapenemase. Second, we did not conduct a drug susceptibility test for tigecycline and colistin. Third, the efflux pump was not analyzed in this study. Fourth, no function assessment of porin mutation was done in this study. Fifth, we defined community- and hospital-acquired CRE according to the previous studies (Garner et al., 1988; Weinstein et al., 1997). Thus, a patient with multiple exposures to the healthcare system can be classified into community-acquired CRE. Sixth, we could not explain the carbapenem-resistant mechanisms underlying three CREc isolates.

## Conclusion

Complete genome sequencing detected that gyrA D87N mutation mediated fluoroquinolone resistance in all the CREc isolates in this study, suggesting a common mechanism. We also identified that OmpF 264-269 deletions might contribute to carbapenem resistance in CREc isolates. This study further provides insight into the clonal diversity, resistant mechanisms, and virulence factors of CREc strains isolated from patients with bacteremia in Taiwan. In *E. coli*, *Bla*_NDM−1_ mutation can be observed in patients with community-acquired bacteremia and in patients without risk factors for CRE infection. Combined clinical and genomic analyses indicate the highly diverse nature of the CREc in Taiwan.

## Data Availability Statement

The datasets presented in this study can be found in online repositories. The names of the repository/repositories and accession number(s) can be found below: https://www.ncbi.nlm.nih.gov/genbank/, CP084378.

## Author Contributions

W-TY, P-YL, and Y-TH contributed to the conception and design of the study and wrote the first draft of the manuscript. W-TY and P-YL contributed to the collection of clinical isolates. P-YL and Y-TH contributed to the experiments. Y-TH performed the bioinformatic analysis. All authors contributed to revising the manuscript and have approved the submitted version.

## Funding

Y-TH was supported in part by the Ministry of Science and Technology (109-2221-E-194-038-MY3). P-YL was supported in part by the Ministry of Science and Technology (110-2314-B-075A-011) and the Taichung Veterans General Hospital (TCVGH-1113901C, TCVGH-1113901D, TCVGH-NK1109004, TCVGH-NK1099002, and TCVGH-1093902D).

## Conflict of Interest

The authors declare that the research was conducted in the absence of any commercial or financial relationships that could be construed as a potential conflict of interest.

## Publisher's Note

All claims expressed in this article are solely those of the authors and do not necessarily represent those of their affiliated organizations, or those of the publisher, the editors and the reviewers. Any product that may be evaluated in this article, or claim that may be made by its manufacturer, is not guaranteed or endorsed by the publisher.

## Abbreviations

CA: community-acquired
CAD: coronary artery disease
CAZ: ceftazidime
Cefo/sulb: Cefoperazone/sulbactam
CEX: cephalexin
CFPM: cefepime
CJD: Creutzfeldt-Jakob disease
CKD: chronic kidney disease
CLSI: Clinical and Laboratory Standards Institute
CNSE: carbapenem non-susceptible Enterobacteriaceae
CP-CRE: carbapenemase-producing carbapenem-resistant Enterobacteriaceae
CP-CREc: carbapenemase-producing carbapenem-resistant *E. coli*
DM: diabetes mellitus
DOR: doripenem
ESRD: end-stage renal disease
ETP: ertapenem
ExPEC: extra-intestinal pathogenic *E. coli*
FLO: flomoxef
HA: hospital-acquired
HBV: hepatitis B virus
HCC: hepatocellular carcinoma
HCV: hepatitis C virus
HTN: hypertension
IAI: intra-abdominal infection
LVX: levofloxacin
MIC: minimum inhibitory concentration
Non-CP-CRE: non-carbapenemase-producing carbapenem-resistant Enterobacteriaceae
Non-CP-CREc: non-carbapenemase-producing carbapenem-resistant *E. coli*
PCKD: polycystic kidney disease
UPEC: uropathogenic *E. coli*
UTI: urinary tract infection.
