# Corrigendum: Comparative genomics revealed fluoroquinolone resistance determinants and OmpF deletion in carbapenem-resistant *Escherichia coli*

**DOI:** 10.3389/fmicb.2022.1094324

**Published:** 2022-11-24

**Authors:** Wan-Ting Yang, I-Ju Chiu, Yao-Ting Huang, Po-Yu Liu

**Affiliations:** ^1^Division of Infection, Department of Internal Medicine, Taichung Veterans General Hospital, Taichung, Taiwan; ^2^Department of Computer Science and Information Engineering, National Chung Cheng University, Chia-Yi, Taiwan; ^3^Ph.D. Program in Translational Medicine, National Chung Hsing University, Taichung, Taiwan; ^4^Rong Hsing Research Center for Translational Medicine, National Chung Hsing University, Taichung, Taiwan

**Keywords:** carbapenem-resistant, whole-genome sequencing, *Escherichia coli*, carbapenemase, virulence, epidemiology

In the published article, there was an error in Table 2 as published. Column titles for TEM, CMY, and CTX were displayed as “AmpC,” “ESBL,” and “Broad-Spectrum Beta-Lactamase.” The correct labels are “Broad-Spectrum Beta-Lactamase” for the TEM columns, “AmpC” for the CMY columns, and “ESBL” for the CTX columns. In addition, the Elppa 2 CTX-M-5 block was displayed as “black.” The correct indication is “white.” The corrected Table 2 and its caption appear below.

**Table 2:**
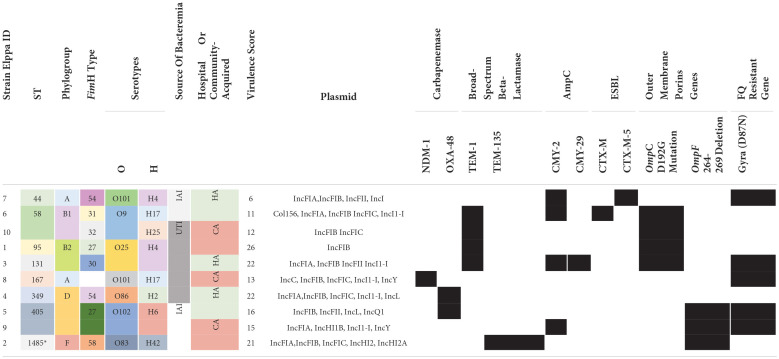
Genetic diversity, source of bacteremia, and antibiotic-resistant mechanisms identified in the *E. coli* strains.

Virulence score is the number of virulence genes that were detected in an isolate. CA, community-acquired; FQ, fluoroquinolone; HA, hospital-acquired; IAI, intraabdominal infection; ST, sequence type; UTI, urinary tract infection.

The authors apologize for these errors and state that this does not change the scientific conclusions of the article in any way. The original article has been updated.

## Publisher's note

All claims expressed in this article are solely those of the authors and do not necessarily represent those of their affiliated organizations, or those of the publisher, the editors and the reviewers. Any product that may be evaluated in this article, or claim that may be made by its manufacturer, is not guaranteed or endorsed by the publisher.

