# Supplementary material for: Comparative Genomics Revealed Fluoroquinolone Resistance Determinants and OmpF Deletion in Carbapenem-Resistant Escherichia coli
Source: Front Microbiol. 2022 Apr 18;13:886428. doi: 10.3389/fmicb.2022.886428 (PMC9062692; doi:10.3389/fmicb.2022.886428)

Supplementary Table 1. The minimal inhibitory concentration (mg/L) of carbapenem-resistant *E. coli* isolates in this study.

Non-CP-CREc, non-carbapenemase-producing carbapenem-resistant *E. coli;* CP-CREc, carbapenemase-producing carbapenem-resistant *E. coli;* Pip/Tazo, piperacillin/tazobactam; TMP/SMX, trimethoprim/sulfamethoxazole.

|  | **Strain ID** | **Ceftriaxone** | **Ceftazidime** | **Cefepime** | **Ceftazidime-avibactam** | **Amikacin** | **Gentamicin** | **Ciprofloxacin** | **Pip/Tazo** | **TMP/SMX** | **Ertapenem** | **Imipenem** |
| --- | --- | --- | --- | --- | --- | --- | --- | --- | --- | --- | --- | --- |
| **Non-CP-CREc** | **Elppa 1** | R<=1 | R>=64 | R>=64 | S | S=16 | R>=16 | S=0.5 | R>=128 | R>=320 | R>=8 | R>=16 |
|  | **Elppa 2** | R<=1 | R>=64 | R>=64 | S | S=16 | I=8 | S<=0.25 | R>=128 | R>=320 | R>=8 | R>=16 |
|  | **Elppa 3** | R>=64 | R>=64 | R=16 | S | S<=2 | S<=1 | R>=4 | R>=128 | **S<=20** | R>=8 | R>=16 |
|  | **Elppa 6** | R>=64 | R>=64 | R>=32 | S | S=4 | R>=16 | S<=0.25 | R>=128 | R>=320 | R>=8 | R=4 |
|  | **Elppa 7** | R>=64 | R>=64 | R>=32 | S | **S=16** | R>=16 | R>=4 | R>=128 | R>=320 | R>=8 | R=4 |
|  | **Elppa 9** | R>=64 | R>=64 | D=4 | S | S<=2 | R>=16 | R>=4 | R>=128 | S<=20 | R>=8 | R=4 |
|  | **Elppa 10** | R<=1 | R>=64 | R>=64 | S | S=16 | I=8 | S<=0.25 | R>=128 | R>=320 | R>=8 | R>=16 |
| **CP-CREc** | **Elppa 4** | R>=64 | I=8 | R=16 | S | S<=2 | S<=1 | S<=0.25 | R>=128 | R>=320 | R=2 | R>=16 |
|  | **Elppa 5** | R>=64 | I=8 | R>=32 | S | I=32 | R>=16 | R>=4 | R>=128 | S<=20 | R>=8 | R=4 |
|  | **Elppa 8** | R>=64 | R>=64 | R>=32 | R | S<=2 | R>=16 | R>=4 | R>=128 | R>=320 | R>=8 | R=8 |

Supplementary Table 2. The mean number of virulence factors and mean Pitts bacteremia score between non-carbapenemase-producing and carbapenemase-producing carbapenem-resistant *E. coli* isolates.

Non-CP-CREc, non-carbapenemase-producing carbapenem-resistant *E. coli;* CP-CREc, carbapenemase-producing carbapenem-resistant *E. coli*

|  | Non-CP-CRE | CP-CRE |
| --- | --- | --- |
| Mean virulence factors | 16.14 | 17 |
| Mean Pitts bacteremia score | 0.71 | 6.3 |

Supplementary Table 3. Genome assembly statistics of the 10 *E coli* isolates sequenced in this study.

| Isolate | Accession | Num. of Chromosomes | Num. of Plasmids | Genome Size | Sequencing Coverage |
| --- | --- | --- | --- | --- | --- |
| elppa1 | CP083534 | 1 | 2 | 5165161 | 113 |
| elppa2 | CP083530 | 1 | 3 | 4904554 | 60 |
| elppa3 | CP083517 | 1 | 11 | 4912152 | 114 |
| elppa4 | CP083512 | 1 | 4 | 5033175 | 114 |
| elppa5 | CP083503 | 1 | 8 | 5117723 | 59 |
| elppa6 | CP083497 | 1 | 5 | 4686611 | 108 |
| elppa7 | JAIQZL000000000 | 1 | 6 | 4816706 | 124 |
| elppa8 | CP083491 | 1 | 5 | 4828629 | 77 |
| elppa9 | CP083481 | 1 | 9 | 5029427 | 177 |
| elppa10 | CP083478 | 1 | 2 | 4755062 | 103 |

**Supplementary Figure 1. Phylogenetic tree of OmpC in tested strains.**


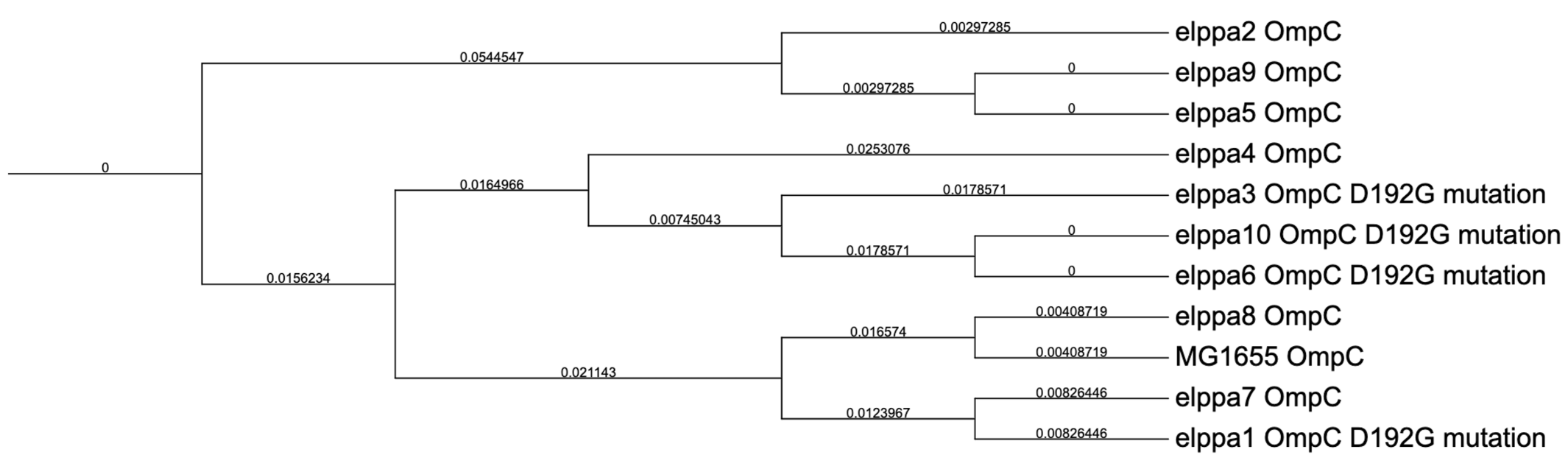

Supplement: Supplementary file 1 [file Data_Sheet_1.docx]
